# Bidirectional Associations Between Alcohol Drinking and Depressive Symptoms Among US Adults Aged 50 to 75: The US Health and Retirement Study

**DOI:** 10.3390/healthcare13010053

**Published:** 2024-12-31

**Authors:** Xinhua Yu, Easter P. Gain, Mark’Quest J. Ajoku, Satish K. Kedia

**Affiliations:** 1 Division of Epidemiology, Biostatistics and Environmental Health, School of Public Health, University of Memphis, Memphis, TN 38152, USA; egain@memphis.edu (E.P.G.); majoku@memphis.edu (M.J.A.); 2 Division of Social Behavioral Sciences, School of Public Health, University of Memphis, Memphis, TN 38152, USA; skkedia@memphis.edu

**Keywords:** alcohol drinking, depression, middle-aged adults, bidirectional association, causal association

## Abstract

**Background/Objectives**: Low or moderate alcohol drinking may reduce the risk of depression, but depression may induce alcohol drinking. However, the bidirectional associations between alcohol drinking and depression were inconsistent, and many prior analyses were not properly conducted. This study explored the within-individual bidirectional associations between alcohol drinking and depressive symptoms under a causal analytic framework. **Methods**: Using data for the baby boomer cohorts (born between 1948 and 1965) from the Health and Retirement Study (HRS), we employed the unit fixed-effect models with lagged measures to examine the within-individual bidirectional associations between the number of alcohol drinks per week and the changes in the eight-item Center for Epidemiological Studies-Depression (CES-D) scores. **Results**: Among 11,057 participants at baseline, about 48% were drinkers and 19% had a CES-D ≥4, i.e., at a high risk of depression. Among male low/moderate drinkers, increasing alcohol drinking between consecutive visits was significantly associated with a decrease in depression scores after adjusting for prior alcohol drinking (−0.15 points per 7 drinks/week increase, *p* = 0.009). Conversely, among male drinkers and female heavy drinkers, increasing depression scores between visits increased alcohol drinking after adjusting for prior depression scores (ranging from 0.22 to 0.79 drinks/week per 1 point increase of depression score, all *p* values < 0.01). **Conclusions**: The bidirectional associations between alcohol drinking and depressive symptoms were evident only among male drinkers, and alcohol drinking should not be recommended as a solution for preventing or relieving depressive symptoms. **Limitations**: Measures of alcohol drinking and depression were coarse, and the study cohorts were limited to the US baby boomer generation. Generalizing findings to other populations should be cautious.

## 1. Introduction

Depression is a leading cause of disease burden globally [1,2,3]. About 10–20% of people, regardless of age, reported experiencing depressive symptoms at any year [4]. Preventing depression would significantly reduce the burden of depression in the population and improve the quality of life among affected individuals. On the other hand, alcohol drinking is culturally acceptable and often considered beneficial to health (as evident in the phrase “toast to your health”), and half of older adults in the US drink some amount of alcohol [5]. In addition to social drinking, many older adults believe the purported benefits of low/moderate drinking in reducing the risks of heart diseases and diabetes [1,6,7,8,9,10,11,12]. Several studies have also shown that low/moderate alcohol drinking may be related to a lower risk of depression [13,14,15,16,17,18,19]. For example, a secondary analysis of the large PREDIMED trial (Prevention with Mediterranean Diet) showed that moderate alcohol drinking (5–15 g/day, or 1 drink/day) was associated with a lower risk of incident depression among people aged 55 or older over seven years of follow up [14]. A pooled analysis based on three large British, American, and Chinese cohorts of middle-aged or older participants also found that low to moderate drinking was significantly associated with a lower incidence of depressive symptoms compared to no drinking [17]. A J-shaped or U-shaped association between the amount of alcohol drinking and the risk of depression or depression symptoms was reported in a recent meta-analysis [20]. However, a pooled study of European cohorts did not find a reduced risk of depression by moderate alcohol drinking [21]. Nonetheless, excessive or heavy drinking were consistently shown to increase the risk of depression [20,22,23,24,25,26,27,28]. In addition, people with depression may increase the amount of alcohol drinking [22,23] as a coping strategy for relieving tension and stress, i.e., the “self-medication” hypothesis [22,29,30,31]. On the other hand, depressed people may reduce drinking due to lack of social interactions with others. 

The possible bidirectional associations between alcohol drinking and depression were explored using longitudinal studies with mixed results [32,33,34]. One recent study used cross-lagged panel analysis to explore the bidirectional associations between depression and alcohol drinking (also smoking and other substance use) explicitly [35] but did not find any bidirectional associations. Another study found that alcohol use disorder increased the risk of depression but not the converse [36].

Most previous studies examining the bidirectional associations did not distinguish between-individual and within-individual effects. For example, traditional cross-lagged panel analysis combined the effects from both within-individual and between-individuals, which may be subjected to between-individual confounding [37]. In addition, the current understanding of causal inference based on the potential outcome framework [38] emphasizes the within-individual differences. Recently, fixed-effect models with lagged measures of both outcomes and predictors were proposed for better causal inferences [39]. The fixed-effect model can explicitly estimate the impact of within-individual changes in exposure on the within-individual changes in outcomes.

In this study, we set to explore the within-individual bidirectional associations between alcohol drinking and self-reported depressive symptoms in a longitudinal study of US middle-aged adults to young older adults (aged 50 to 75). We hypothesize that there are bidirectional associations between alcohol drinking and depression longitudinally.

## 2. Materials and Methods

### 2.1. Study Population

This study used data from the Health and Retirement Study (HRS), a longitudinal survey of over 37,000 US adults aged 50 or older since 1992 [40]. Details of the study design, survey instruments, and data documents are available on the website [41] hosted by the University of Michigan. We used the RAND version of the harmonized file that included data for all visits until 2020. Since the data were publicly available, no additional ethical approval was needed from the authors’ institutions.

In the HRS, participants were recruited through a complex sampling design and surveyed every two years with over 80% response rates. New cohorts have been added every six years, and since 1998, survey instruments remained consistent across visits. The current study included only baby boomer generations who were recruited at the visit 7 in 2004 (early baby boomers, born between 1948 and 1953, EBB, n = 4785), 2010 (mid-baby boomers, born between 1954 and 1959, MBB, n = 5113), and 2016 (late baby boomers, born between 1960 and 1965, LBB, n = 4597). We chose these cohorts because the measurements of depression and alcohol drinking were more consistent in these new cohorts, and cohort effects were avoided due to their similar life experiences.

**Figure A1:**
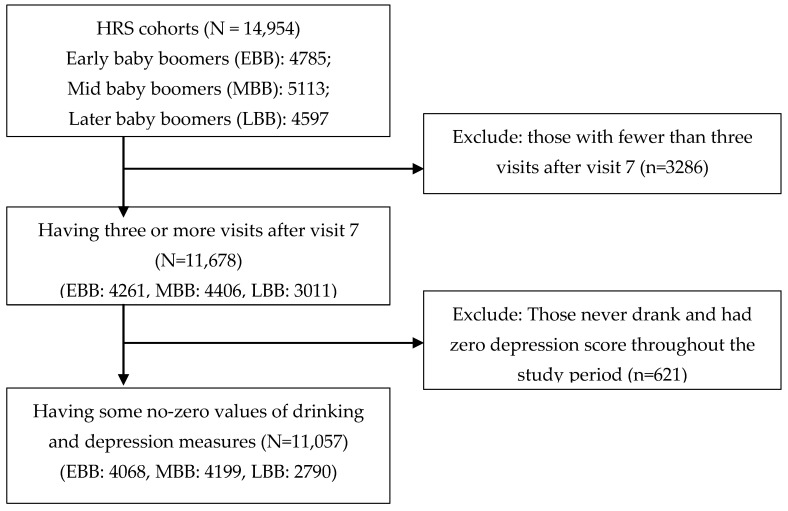
Flowchart for assembling the study sample from the Health and Retirement Study.

Furthermore, because we would employ fixed-effect models with lagged measures, we required all participants to have at least three visits (n = 11,678). Since participants who never drank and had a zero CES-D score across all visits would not contribute to the model estimation in the fixed-effect models, they were dropped from the final analysis (n = 621), resulting in 11,057 participants in the final dataset (EBB: 4068, MBB: 4199, LBB: 2790) (see Appendix A Figure A1 for the flowchart).

### 2.2. Measuring Alcohol Drinking

In all visits, participants were asked whether they ever drank any alcohol, and if they did, the average number of days per week they drank and the number of drinks per day when they drank. They were converted into the number of drinks per week. Participants were grouped into nondrinkers (0 drink/week), low/moderate drinkers (≤14 drinks/week for males, ≤7 drinks/week for females), and heavy drinkers (>14 drinks/week for males, and >7 drinks/week for females). No types of drinks were recorded, and binge drinking was not included in the RAND file.

### 2.3. Measuring Depressive Symptoms

Depressive symptoms were measured using the abridged 8-item Center for Epidemiological Studies-Depression (CES-D) measure. If participants answered “yes” to “much of time” during the past week on the follow six items: “felt depressed”, “felt everything an effort”, “sleep was restless”, “could not get going”, “felt lonely”, and “felt sad”, a value of 1 was assigned to the item, while the two positive items, “enjoy life” and “was happy”, were reverse coded as 0 for “yes”. The CES-D score was the sum of these eight items, ranging from 0 to 8. A CES-D score ≥4 was used to indicate a high risk of depression [42].

### 2.4. Measuring Other Characteristics

HRS measured many socioeconomic factors, health conditions, employment status, and other psycho-social factors during each visit. We included age of participants, race (Black, White, and others), sex (male and female), education (less than high school, high school, and some college or more), marital status (married/partnership, divorced/separated, widowed/single/other), household income (<$30,000, $30,000–50,000, $50,000–70,000, and $70,000 or more), ever had a comorbidity (diabetes, heart disease, stroke, and cancer) and had the comorbidity since last visit, which were all coded as “yes/no”. Employment status (including retirement status) was coded as current working (yes/no) during each visit. Since 2004, the frequencies of physical activities were measured for vigorous, moderate, and light activities separately. They were coded as none, 1–3 times per month, and 1 or more times per week.

### 2.5. Statistical Analysis

The baseline characteristics of participants were presented with descriptive statistics. Comparisons were based on *t*-tests for continuous variables and χ^2^ tests for categorical variables. In addition, although males tend to have a higher level of alcohol drinking than females, the health impact of alcohol drinking may be larger among females and males for the same amount of alcohol drinking. On the other hand, females are more likely to report depressive symptoms than males. Therefore, since both alcohol drinking behavior and risk of depression differ between males and females, all analyses were performed for males and females separately. Statistical analyses were weighted by baseline sampling weights to account for the survey design.

In this study, we employed the unit fixed-effect model with lagged measures of both outcome and exposure (similar to the autoregressive distributed lag model) to estimate the bidirectional associations between alcohol drinking and depression score. Specifically, let Y_i,t_ and X_i,t_ be the outcome and key predictor at the visit t for individual i, and ΔX_i,t_ = X_i,t_ − X_i,t−1_ be the difference between the visit t and t − 1 (one interval before) for the predictor; then, the longitudinal fixed-effect model is [39]

**Figure A2:**
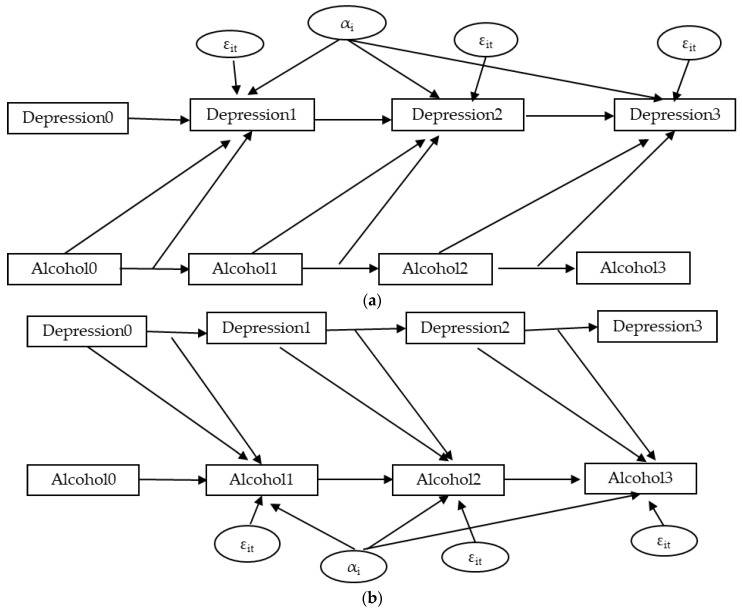
Diagrams for the lagged fixed-effect model with four waves. (**a**) Alcohol drinking predicting depression, (**b**) depression predicting alcohol drinking.

Y_i,t_ = α_i_ + β*ΔX_i,t_ + γ*X_i,t−1_ + ρ*Y_i,t−1_ + λ*Z_i,t_ + ε_i,t_

where α_i_ is the individual specific intercept that absorbs time-invariant confounding effects, β is the average effect on the current Y at the visit t by the change in X from visit t − 1 to t, γ is the predicting effect of prior X from the visit t − 1, ρ is the one-lagged autoregressive coefficient of Y from the visit t − 1, λ is the effect of the time-varying confounder Z_i,t_, and ε_i,t_ is the individual- specific and time-specific random error. Note that including both X_i,t−1_ and ΔX_i,t_ in the model is equivalent to modeling both X_i,t−1_ and X_i,t_, which was suggested for models with lagged dependent variables [43] and also appropriate with ordinary least square estimation [44]. Furthermore, since previous studies showed that both nondrinkers and heavy drinkers might be at a higher risk of depression, we included interaction terms between (ΔX_i,t_, X_i,t−1_, Y_i,t−1_) and the baseline drinking levels (no drinking, low/moderate drinking, and heavy drinking) in the above model, i.e., modeling different slopes for different baseline drinking levels (see Appendix A Figure A2 for model diagrams).

The fixed-effect models explicitly estimate the within-individual effects and account for confounders that do not change over time (e.g., race or education level). However, time-varying confounders such as age, smoking behavior, physical exercise, marital status, income, employment status, and comorbidities may change during the follow-up visits. These time-varying confounders were easily adjusted in the models (the term Zs). Finally, we considered the age-adjusted model as the basic model because the age axis was of practical importance, not the calendar time.

In the primary analyses, models were performed separately for depression or alcohol drinking analyzed as continuous outcomes with and without adjustment for various covariables. Additionally, we performed sensitivity analysis by dichotomizing CES-D scores at the cutoff point 4 and categorizing alcohol drinking as never, low/moderate, and heavy drinkers, and fitted with fixed-effect logistic regressions. However, since fixed-effect models discard all observations that had no changes in both outcome and predictor status, fixed-effect logistic regressions had significantly low statistical power. Finally, we also explored difference-in-difference models instead of lagged autoregressive outcomes. Conclusions were consistent with the primary analyses.

All the tests were two-sided with a *p* value < 0.05 considered statistically significant. SAS 9.4 and Stata 16.1 were used for the analysis.

## 3. Results

The unweighted distributions of participant’s characteristics at baseline were presented in Table 1 by baseline drinking status. Of 11,057 participants, 52.4% were nondrinkers, 38% were low/moderate drinkers, and 10% were heavy drinkers. The mean age was 54 years old (standard deviation: 2.6). Around 19% of the population had a CES-D ≥4, i.e., at a high risk of depression. Notably, those low/moderate drinkers had the lowest percentage of a CES-D ≥4 (13.8%) compared with 22.5% among nondrinkers and 19.1% among heavy drinkers. Socioeconomic characteristics were significantly different across drinking status. Low/moderate drinkers were more likely to be Whites, with higher education levels or higher household income, married/living with partners, or currently employed, compared to other drinking status. In addition, those with comorbidities were less likely to drink than those without. Interestingly, 28% of participants engaged in vigorous physical activities weekly at the baseline, and those who drank were more likely to be physically active.

**Figure 1:**
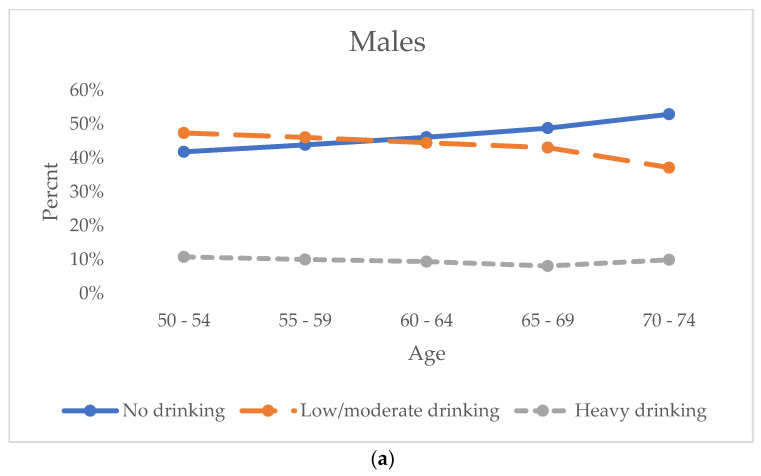

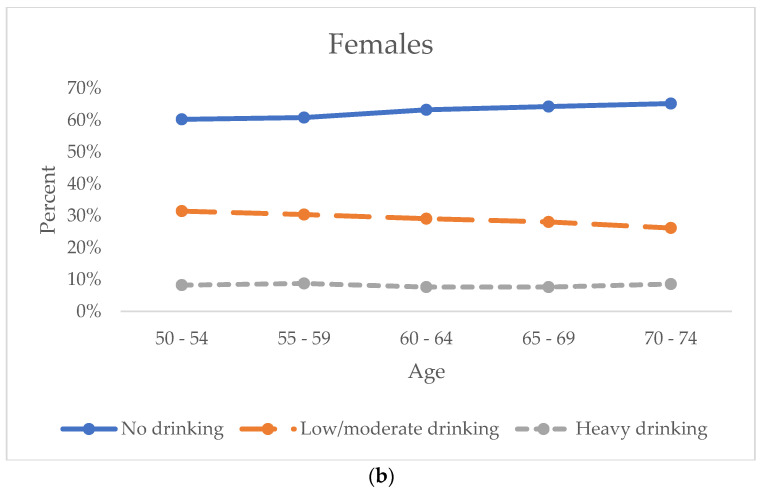
Trend of alcohol drinking among males (**a**) and females (**b**) by age groups over the follow –up among baby boomer generation in the Health and Retirement Study (2004–2020).

**Figure 2:**
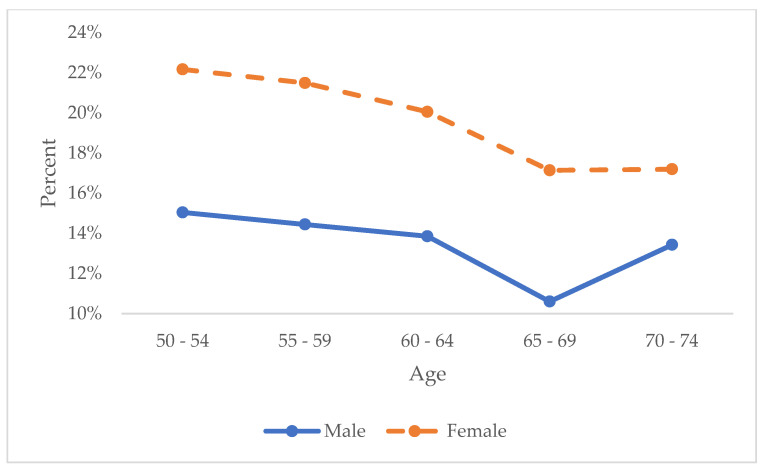
Trend of at high-risk of depression (CES-D ≥ 4) among males and females by age groups over the follow–up among baby boomer generation in the Health and Retirement Study (2004–2020). CES-D: Center for Epidemiologic Studies Depression, 8-item short version.

We explored the patterns of alcohol drinking (Figure 1a,b) and the proportions of having a high risk of depression (CES-D ≥ 4) (Figure 2) by age groups for males and females separately. The prevalence of heavy drinking remained stable across age groups for both males and females, while the prevalence of low/moderate drinking declined with age, resulting in an increase in the prevalence of nondrinkers. In addition, the prevalence of having a high risk of depression decreased for both males and females during the middle age, and males had higher prevalence than females. On the other hand, the gender differences in the prevalence of having high risk of depression were significantly larger before age 70, and after that, the prevalence of having a high risk of depression increased among females, and the gender difference reduced (Figure 2).

Table 2 presents the estimated within-individual effects between alcohol drinking and self-reported depressive symptoms after adjusting for time-varying confounders. The bidirectional associations were more evident among male drinkers than females. A higher level of drinking at the prior visit was related to increased depression scores between visits among male nondrinkers and low/moderate drinkers. On the other hand, increasing drinking between visits was associated with decreases in depression scores, especially among male low/moderate drinkers. There were no such patterns among females.

The association between depression score changes and alcohol drinking has different patterns. Among males, higher depression scores at the prior visit were associated with increased drinking at the follow-up visit, especially among male heavy drinkers. Furthermore, an increase in depression scores between visits led to a significant increase in alcohol drinking among male low/moderate and heavy drinkers, which was also true for female heavy drinkers.

## 4. Discussion

Among US adults aged from 50 to 75 in the Health and Retirement Study, we found bidirectional associations between moderate alcohol drinking and self-reported depressive symptoms among male drinkers. Increasing drinking between visits might be associated with a reduced risk of depression among male drinkers; however, we did not find a similar pattern among females. On the other hand, an increase in depression scores between visits was significantly associated with an increase in alcohol drinking among male low/moderate and heavy drinkers and also among female heavy drinkers. Our exploratory results provided further evidence for elucidating the bidirectional causality between alcohol consumption and depression.

Our findings were consistent with the protective effects of past low/moderate alcohol drinking on the risk of depression commonly found in cross-sectional studies and some longitudinal studies [13,14,15,16,17,18,19,20]. However, most previous studies used population average models, while our current study examined within-individual effects. As shown in the current study, there was an inverse association between low/moderate alcohol drinking and depression at baseline, and in the within-individual effect models, increasing alcohol drinking might reduce the risk of depression among male low/moderate drinkers.

Our results might also support the “self-medication” hypothesis in which the occurrence of depression may lead to increased alcohol drinking [22,29,30,31]. A previous study showed that major depression could lead to an increased risk of alcohol abuse and other substance abuse [23], but not all studies demonstrated a bidirectional association between alcohol drinking and depression [32,33,34]. For example, one recent cross-lagged panel analysis did not find bidirectional associations between depression and alcohol drinking (also smoking and other substance use) [35], and another recent study found that the risk of depression was increased with alcohol use disorders, but not the converse [36]. However, our findings suggest that the inducing effects from depression to alcohol drinking might present among male drinkers and female heavy drinkers.

As people age, the alcohol metabolic rate slows down and becomes less tolerable to alcohol than before. Therefore, erroneous beliefs such as drinking to prevent depression or reduce depressive symptoms may harm health among older adults. The risk limit of alcohol drinking may be lower than previously assumed. For example, the risk of overall mortality and cardiovascular diseases started increasing significantly after 100 g of alcohol per week for males, which renders to 7 drinks/week with the US standard of 14 g/drink [12]. This is only half of the recommendation by the National Institute on Alcohol Abuse and Alcoholism. In addition, the protecting effects of diabetes and heart diseases may be overshadowed by the risk of hypertension, stroke, liver diseases and cancer, leading to a net benefit of zero for most elderly adults [1,2,3,24,45,46].

The main strength of our study was the use of unit fixed-effect models with lagged measures to examine the within-individual differences, which is more aligned with the causal inference framework [38]. However, the main disadvantage of the fixed-effect model is the reduced power to detect statistical significance, as the magnitude of differences within individuals is typically small in socio-behavioral studies. Other methods such as marginal structure models [47], cross-lagged panel analysis [37,48], and dynamic models [49,50] based on structural equation models could be used. Some of them combined effects from both within-individual and between-individuals. However, between-individual differences tend to be confounded by many known and unknown confounders between individuals, while fixed-effect models can be viewed as a self-matched analysis, thus adjusting for both known and unknown time-invariant confounders.

Another major strength of the current study was the restricted study population to late middle-age and early old adults, which is a period with many changes in employment, financial prospects, and health conditions. In fact, over 20% of participants had four or more depressive symptoms in their early 50s, suggesting that targeted behavioral interventions would yield the most cost-effectiveness in preventing depression.

One limitation was that HRS used coarse measures of alcohol drinking without detailed information about types of drinks and binge drinking. Depressive symptom score was measured with the eight-item CES-D and coded as yes/no instead of the Likert scale (0–4) in the standard CES-D. Although CES-D has been shown to be highly valid in community surveys, it is far from perfect. The semi-quantitative nature of the depression assessment may be less sensitive to the changes in depression. More extensive measures such as the Beck Depression Inventory (BDI) or Hamilton Depression Rating Scale (HAM-D) for measuring depression may provide more detailed and accurate measures. Similarly, HRS used simple frequency questionnaires for measuring alcohol drinking. Other more extensive measures such as the Alcohol Use Disorders Identification Test (AUDIT) may be better for evaluating drinking behaviors. In addition, no clinical information about depression medications and diagnosis was available, which might underestimate the true level of depression in the population. The study participants who attended the visits were unlike to have clinically diagnosed major depression disorder. We also did not include many social factors such as social network, social isolation, employment changes, and financial volatility in the analysis. These factors can play important roles in affecting both alcohol drinking behavior and depression. In addition, our study is a secondary data analysis of an observational study. Although our analytical methods were based on causal inference framework, it is still not possible to firmly establish any causal association. Our study should be considered exploratory. Finally, our analysis was limited to the baby boomer generation in the US. Thus, generalizing our findings to other populations should be cautious. Cultural norms, generation differences and other social differences may affect the association between alcohol drinking and depression.

## 5. Conclusions

Our study provided strong evidence for bidirectional associations between alcohol drinking and self-reported depressive symptoms among middle-aged male drinkers based on rigorous causal analytic methods. However, we do not recommend alcohol drinking to prevent or relieve depressive symptoms given the net harmful effects of alcohol drinking among older adults. Further intervention studies could explore whether other behavioral changes instead of alcohol drinking can reduce depressive symptoms.

## Figures and Tables

**Table 1:** Characteristics of participants at baseline for the baby boomer cohorts in the Health and Retirement Study (first interviewed in 2004, 2010, and 2016).

		Total	No Drinking	Low/Moderate Drinking	Heavy Drinking
All		11,057	5790 (52.4%)	4210 (38.1%)	1057 (9.6%)
Age (mean/SD)		54.1 (2.6)	54.1 (2.7)	54.0 (2.6)	54.0 (2.6)
CES-D score (mean/SD)		1.8 (2.2)	2.1 (2.3)	1.4 (1.9)	1.8 (2.2)
	0	3855 (34.9%)	1676 (28.9%)	1815 (43.1%)	364 (34.4%)
	1–3	5116 (46.3%)	2812 (48.6%)	1813 (43.1%)	491 (46.4%)
	>=4	2086 (18.8%)	1302 (22.5%)	582 (13.8%)	202 (19.1%)
Alcohol drinks/week (mean/SD)		3.4 (7.0)	0 (0)	4.1 (3.2)	20.5 (11.3)
Gender					
	Male	4781 (43.2%)	2033 (35.1%)	2226 (52.9%)	522 (49.4%)
	Female	6276 (56.8%)	3757 (64.9%)	1984 (47.1%)	535 (50.6%)
Race					
	White	6506 (59.1%)	3181 (55.2%)	2638 (63.0%)	687 (65.4%)
	Black	2877 (26.2%)	1600 (27.8%)	1040 (24.8%)	237 (22.6%)
	Others	1616 (14.7%)	977 (17.0%)	512 (12.2%)	127 (12.1%)
Education					
	Less than high school	1729 (15.6%)	1128 (19.5%)	426 (10.1%)	175 (16.6%)
	High school graduates	3307 (29.9%)	1808 (31.2%)	1150 (27.3%)	349 (33.0%)
	some college or more	6021 (54.5%)	2854 (49.3%)	2634 (62.6%)	533 (50.4%)
Marital status					
	Married/partnership	7423 (67.1%)	3726 (64.4%)	2981 (70.8%)	716 (67.7%)
	Divorce/separated	2627 (23.8%)	1497 (25.8%)	881 (20.9%)	249 (23.6%)
	Widowed/single/other	1007 (9.1%)	567 (9.8%)	348 (8.3%)	92 (8.7%)
Household income ($)					
	<30,000	2601 (23.5%)	1649 (28.5%)	710 (16.9%)	242 (22.9%)
	30,000–50,000	2764 (25.0%)	1652 (28.5%)	869 (20.7%)	243 (23.0%)
	50,000–70,000	2900 (26.2%)	1454 (25.1%)	1178 (28.0%)	268 (25.3%)
	70,000+	2792 (25.3%)	1035 (17.9%)	1453 (34.5%)	304 (28.8%)
Employed		7648 (70.0%)	3693 (64.5%)	3210 (77.3%)	745 (71.4%)
Vigorous physical activity					
	None	5475 (49.5%)	3333 (57.6%)	1658 (29.4%)	484 (45.8%)
	1–3/month	2482 (22.5%)	1116 (19.3%)	1121 (26.6%)	245 (23.2%)
	>=1/week	3106 (28.0%)	1341 (23.2%)	1431 (34.0%)	328 (31.0%)
Moderate physical activity					
	None	1638 (14.8%)	1147 (19.8%)	376 (8.9%)	115 (10.9%)
	1–3/month	3475 (31.4%)	1846 (31.9%)	1298 (30.8%)	331 (31.3%)
	>=1/week	5944 (53.8%)	2797 (48.3%)	2536 (60.2%)	611 (57.8%)
Light physical activity					
	None	660 (6.0%)	428 (7.4%)	176 (4.2%)	56 (5.3%)
	1–3/month	3387 (30.6%)	1851 (32.0%)	1231 (29.2%)	305 (28.9%)
	>=1/week	7010 (63.4%)	3511 (60.6%)	2803 (66.6%)	696 (65.9%)
Diabetes		1826 (16.5%)	1208 (20.9%)	504 (12.0%)	114 (10.8%)
Heart disease		1116 (10.1%)	676 (11.7%)	352 (8.4%)	88 (8.4%)
Stroke		396 (3.6%)	266 (4.6%)	104 (2.5%)	26 (2.5%)
Cancer		608 (5.5%)	347 (6.0%)	210 (5.0%)	51 (4.8%)

Note: 1. CES-D: Center for Epidemiologic Studies Depression, 8-item short version. 2. SD: standard deviation. 3. None: zero drinks/week; Low/moderate drinking: ≤14 drinks/week for males, ≤7 drinks/week for females; Heavy drinking: >14 drinks/week for males, >7 drinks/week for females.

**Table 2:** Bidirectional associations between alcohol drinking and CES-D score among the baby boomer generations in the Health and Retirement Study (2004–2020), by gender.

			Males		Females	
Outcome	Predictor	Baseline Drinking Status	Coefficient (95% CI)	*p*-Value	Coefficient (95% CI)	*p*-Value
Change in depression score between visits						
	Drinks at the prior visit	None	**0.16 (0.02, 0.31)**	**0.023**	−0.13 (−0.33, 0.07)	0.201
		Low/moderate	**0.16 (0.01, 0.30)**	**0.038**	0.08 (−0.05, 0.21)	0.218
		Heavy	0.03 (−0.10, 0.16)	0.664	0.10 (−0.02, 0.21)	0.108
	Change in drinks between visits	None	−0.08 (−0.19, 0.04)	0.193	0.10 (−0.05, 0.25)	0.197
		Low/moderate	**−0.15 (−0.26, −0.04)**	**0.009**	−0.06 (−0.13, 0.02)	0.133
		Heavy	−0.03 (−0.11, 0.06)	0.560	0.00 (−0.08, 0.09)	0.926
Change in alcohol drinking between visits						
	Depression score at the prior visit	None	−0.01 (−0.13, 0.10)	0.803	−0.01 (−0.05, 0.04)	0.732
		Low/moderate	0.25 (−0.04, 0.55)	0.087	0.06 (−0.07, 0.19)	0.382
		Heavy	**1.09 (0.27, 1.91)**	**0.010**	0.53 (−0.03, 1.10)	0.065
	Change in depression scores between visits	None	−0.02 (−0.10, 0.05)	0.530	0.00 (−0.02, 0.02)	0.905
		Low/moderate	**0.22 (0.05, 0.39)**	**0.010**	0.05 (−0.02, 0.12)	0.149
		Heavy	**0.79 (0.23, 1.35)**	**0.006**	**0.51 (0.18, 0.84)**	**0.002**

Note: 1. CES-D: Center for Epidemiologic Studies Depression, 8-item short version. 2. No drinking: zero drinks/week; Low/moderate drinking: ≤14 drinks/week for males, ≤7 drinks/week for females; and Heavy drinking: >14 drinks/week for males, >7 drinks/week for females. 3. Models were adjusted for age, income, marital status, employment status, physical activities, smoking status, and comorbidities.

## Data Availability

Health Retirement Study data are publicly available from https://hrsdata.isr.umich.edu/ (accessed on 30 August 2024).

## Abbreviations

HRS	Health and Retirement Study
EBB	early baby boomers (born between 1948 and 1953)
MBB	mid-baby boomers (born between 1954 and 1959)
LBB	late baby boomers (born between 1960 and 1965)
CES-D	Center for Epidemiological Studies-Depression
